# NS3 Protein from *Rice stripe virus* affects the expression of endogenous genes in *Nicotiana benthamiana*

**DOI:** 10.1186/s12985-018-1014-7

**Published:** 2018-06-25

**Authors:** Gentu Wu, Guixian Zheng, Qiao Hu, Mingge Ma, Mingjun Li, Xianchao Sun, Fei Yan, Ling Qing

**Affiliations:** 1 Chongqing Key Laboratory of Plant Disease Biology, College of Plant Protection, Southwest University, Chongqing, 400716 China; 2 The State Key Laboratory Breading Base for Sustainable Control of Pest and Disease, Key Laboratory of Biotechnology in Plant Protection of MOA of China and Zhejiang Province, Institute of Virology and Biotechnology, Zhejiang Academy of Agricultural Sciences, Hangzhou, 310021 China

**Keywords:** *Rice stripe virus*, NS3, Suppressor of RNA silencing, *Nicotiana benthamiana*, Transcriptomes

## Abstract

**Background:**

*Rice stripe virus* (RSV) belongs to the genus *Tenuivirus*. It is transmitted by small brown planthoppers in a persistent and circulative-propagative manner and causes rice stripe disease (RSD). The NS3 protein of RSV, encoded by the viral strand of RNA3, is a viral suppressor of RNA silencing (VSR). NS3 plays a significant role in viral infection, and NS3-transgenic plants manifest resistance to the virus.

**Methods:**

The stability and availability of NS3 produced by transgenic *Nicotiana benthamiana* was investigated by northern blot analysis. The accumulation of virus was detected by western blot analysis. Transcriptome sequencing was used to identify differentially expressed genes (DEGs) in NS3-transgenic *N. benthamiana.*

**Results:**

When the host plants were inoculated with RSV, symptoms and viral accumulation in NS3-transgenic *N. benthamiana* were reduced compared with the wild type. Transcriptome analysis identified 2533 differentially expressed genes (DEGs) in the NS3-transgenic *N. benthamiana*, including 597 upregulated genes and 1936 downregulated genes. These DEGs were classified into three Gene Ontology (GO) categories and were associated with 43 GO terms. KEGG pathway analysis revealed that these DEGs were involved in pathways associated with ribosomes (ko03010), photosynthesis (ko00195), photosynthesis-antenna proteins (ko00196), and carbon metabolism (ko01200). More than 70 DEGs were in these four pathways. Twelve DEGs were selected for RT-qPCR verification and subsequent analysis. The results showed that NS3 induced host resistance by affecting host gene expression.

**Conclusion:**

NS3, which plays dual roles in the process of infection, may act as a VSR during RSV infection, and enable viral resistance in transgenic host plants. NS3 from RSV affects the expression of genes associated with ribosomes, photosynthesis, and carbon metabolism in *N. benthamiana*. This study enhances our understanding of the interactions between VSRs and host plants.

## Background

*Rice stripe virus* (RSV) belongs to the genus *Tenuivirus*, and is characterized by transovarian transmission in a persistent, circulative-propagative manner by the small brown planthopper (*Laodelphax striatellus*) [1]. Since 2000, RSV has occurred in the Yangtze River region and generally caused a 30–40% reduction in yield in eastern China [2, 3]. RSV comprises four single-stranded RNAs, named RNA1–RNA4 in order of decreasing molecular size, which encode seven proteins with an ambisense expression strategy [4]. RNA1 encodes a protein in the negative-sense (virus-complementary [vc]) strand, which is called RNA-dependent RNA polymerase (RdRp, 337 kDa) [5]. NS2 (silencing suppressor, 22.8 kDa) and NSvc2 (glycoprotein, 94 kDa) are produced from viral-sense RNA2 (vRNA2) and vcRNA2, respectively [6]. RNA3 encodes a nonstructural protein that has been identified as a viral suppressor of RNA silencing (VSR; NS3, 23.9 kDa), and a nucleocapsid protein (CP, 35 kDa)[7], which are derived independently from vRNA3 and vcRNA3 [8]. vRNA4 and vcRNA4 encode a disease-specific protein (NS4) and a movement protein (NSvc4), respectively [9, 10].

RNA silencing is a defense system that interacts with viral genomic RNAs in plants. VSRs from different viral families do not share any obvious sequence similarity, but they generally bind to double stranded RNAs (dsRNAs), such as small interfering RNA (siRNA) duplexes, to suppress RNA silencing and block host RNA silencing pathways by targeting different steps [11–13]. The NS3 protein of *Rice hoja blanca tenuivirus* suppresses RNA silencing in plant and insect hosts by efficiently binding both siRNAs and microRNAs (miRNAs) [14]. P19, the suppressor of *Tomato bushy stunt virus* (TBSV), functions by binding siRNAs to prevent them from forming RNA-induced silencing complex (RISC) [15]. P69, the suppressor of *Turnip yellow mosaic virus* (TYMV), binds dsRNAs to inhibit the initiation of RNA silencing [11, 16, 17]. 2b protein, the suppressor of *Cucumber mosaic virus* (CMV), targets Argonaute (AGO) protein, a core component of RISC [11, 17]. NS3 of RSV, a reported RNA silencing suppressor, suppresses post-transcriptional gene silencing (PTGS) in *Nicotiana benthamiana* through its siRNA and dsRNA binding ability [8].

VSRs may enhance viral pathogenicity and accumulation, but transgenic plants expressing *Tobacco etch virus* (TEV) HC-Pro exhibit enhanced resistance to heterologous TBRV and to the oomycete *Peronospora tabacina* [12]. Transgenic rice expressing RSV NS3 exhibit stronger resistance to rice blast disease (*Magnaporthe grisea oryzae* Guy) than wild-type rice [18]. Furthermore, disease symptoms and viral accumulation are more moderate in transgenic rice than in wild-type rice after RSV inoculation [8]. NS3 not only plays a critical role in combating viral infection at the early stages of disease, but also induces host resistance to the virus and other pathogens. Previous studies showed that NS3-induced miRNA accumulation in rice may lead to enhanced miRNA processing complexes and pathogenicity [19]. The apparent dual roles of NS3 may be further explored using molecular techniques.

To elucidate the underlying mechanisms for host plant responses to RSV infection, we aimed to identify the key genes involved in plant-RSV interactions. Transcriptomic analysis has been a popular approach to explore the unknown players in a wide range of biological processes. In functional genomics, transcriptomic studies typically involve comparisons between biological samples collected under different conditions, and are used to analyze which genes are upregulated or downregulated in response to stresses [20–22]. RSV infection affects expression patterns of plant miR171 and several other miRNAs, and a reduction of osa-miR171b in RSV-infected rice contributes to RSV symptoms [23]. How NS3 enhances host resistance to viral infection remains unclear, but this effect is likely to be mediated by changes in endogenous gene expression. NS3 induces the accumulation of several miRNAs and enhances viral infection and pathogenesis in rice; most of these miRNAs target pivotal genes associated with growth and development or pathogen resistance [19]. This study employed transcriptome analysis and RT-qPCR to uncover the function of NS3 in transgenic *N. benthamiana*. To characterize *N. benthamiana* responses to NS3 at the transcriptome level, we identified genes that were differentially expressed between wild-type and NS3-expressing *N. benthamiana* up to the 6th leaf stage. We aimed to provide novel insights into the mechanisms underlying hosts-VSR interactions.

## Methods

### Plant growth and virus inoculation

Wild-type *N. benthamiana* and NS3-transgenic *N. benthamiana* were grown in a greenhouse under a 16-h light and 8-h dark cycle at 25 °C. NS3-transgenic *N. benthamiana* and wild-type *N. benthamiana* were inoculated with RSV. At 21 days post-infiltration, leaf tissues were collected. Three biological replicates (three plants per replicate) were processed independently.

### Northern blotting

Total RNA was isolated from frozen NS3-transgenic *N. benthamiana* plants, which were not infected with RSV, using Trizol (Invitrogen, Carlsbad, CA, USA) according to the manufacturer’s instructions. Fifty micrograms of DNase-treated total RNA was separated on a 15% agarose gel, and transferred electrophoretically to Hybond-N^+^ membranes using 20× SSC. Membranes were baked at 80 °C for 2 h. DNA oligonucleotides complementary to the putative NS3 sequences were end-labeled with digoxigenin (DIG) using the DIG Oligonucleotide 3′-end Labeling Kit (Roche, 11,585,614,910). Membranes were pre-hybridized for at least 1 h and hybridized overnight at 42 °C using the DIG High Prime Labeling and Detection Starter Kit II (Roche, 11,585,614,910).

### Western blotting

To determine RSV protein accumulation in RSV-infected *N. benthamiana* and RSV-infected NS3-transgenic *N. benthamiana* plants, total proteins were extracted from 0.1 g of plant material in 500 μL of protein extraction buffer. For protein gel blotting, proteins were separated by sodium dodecyl sulfate-polyacrylamide gel electrophoresis (SDS-PAGE) in a 12% gel and transferred to PVDF membranes (BioRad, Hercules, CA, USA). The membranes were blocked for 1 h with 5% milk in PBST buffer at room temperature. After washing, the membranes were incubated with anti-RSV CP antibody overnight at room temperature. Signals were developed in NBT/BCIP buffer (Transgen Biotech, Beijing, China).

### RNA extraction and purification and transcriptome sequencing

Total RNA was extracted from *N. benthamiana* and NS3-transgenic *N. benthamiana* plants uninfected with RSV at the 6th leaf stage using Trizol reagent according to the manufacturer’s instructions (Invitrogen, Carlsbad, CA, USA). RNA was quantified using agarose gel electrophoresis and a NanoDrop. Five RNA samples (CK④, CK⑤, NS3–5①, NS3–6④, and NS3–9④) were sent to the BioMark company for transcriptome sequencing as follows: first, mRNA was purified using poly-T oligo-attached magnetic beads, followed by fragmentation and cDNA synthesis and purification. Library quality was then assessed using the Agilent 2100 system. Libraries were sequenced on the Solexa Illumina HiSeq platform (BioMark Company, Beijing, China). Clean reads were obtained from raw reads by removing reads containing adapter or poly-N, and low-quality reads. The Q20, Q30, GC-content, and sequence duplication level of the clean reads were calculated. Then, clean reads were directly used for further bioinformatics analysis.

### Analysis of differentially expressed genes (DEGs)

The original data from transcriptome sequencing comprised raw reads, and clean reads were obtained after removing adaptor sequences and low-quality reads. All clean reads were mapped to *Solanaceae* reference sequences using bowtie software allowing a 2-bp mismatch. Each gene’s expression level was calculated using reads per kilobase per million mapped reads (RPKM). DEGs were identified by a *P* value ≤0.05 and an expression change of 2-fold or more (|log2Foldchange| ≥ 1) between the two treatments using IDEG6 software. Gene Ontology (GO) annotation and Kyoto Encyclopedia of Genes and Genomes (KEGG) classification were used to determine the main biological functions and pathways related to DEGs. GO was implemented using the GOseq R package, in which gene length bias was corrected, and GO terms with corrected *P* values ≤0.05 were considered significantly enriched in DEGs. GO annotation was performed using the REVIGO web server (http://revigo.irb.hr/). The KEGG classification of the DEGs was performed using the KEGG Mapper Annotate Sequence tool with the BlastKOALA server available on the Kyoto Encyclopedia of Genes and Genomes website (https://www.kegg.jp/kegg/tool/annotate_sequence.html).

### Real-time quantitative PCR (RT-qPCR) validation

Total RNA was extracted from wild-type *N. benthamiana* and NS3-transgenic *N. benthamiana* at the 6th leaf stage using Trizol reagent according to the manufacturer’s instructions (Invitrogen, Carlsbad, CA, USA). cDNA was synthesized from 1 μg of total RNA in a volume of 20 μL using the PrimeScript RT Reagent Kit (TAKARA Bio, RR037A) according to the manufacturer’s instructions. RT-qPCR was performed using the SYBR Green Real-time PCR Master Mix (ToYoBo, JAPAN) with the iQ5 Real-Time PCR system (Bio-Rad, USA) with gene-specific primers (Table 1), each reaction containing 10.0 μL of SYBR Green Real-time PCR Master Mix, 1.0 μL of cDNA, 0.5 μL of (10 pM) primers, and 8.0 μL of water. The expression levels of transcripts are presented relative to the corresponding control samples for each condition. The ubiquitin gene was used as an internal control gene. All RT-qPCR experiments were performed in triplicate.

**Table 1:** Primers used for RT-qPCR

Code of gene	F(5′-3′)	R(5′-3′)
ATP-2-NbS00015227g0002	TATTCAAGCAGTTTATGTACCCGC	TGGGCTGTTTCGTAATGTTTCTC
HIS -0-NbS00000634g0101	AACAGCACGTAAATCCACTGGAG	AACCCACCAAGTAAGCCTCTG
HIS- 2-NbS00002567g0001	ATGGCGAGAACCAAACAAACAG	TCCTGCGCTATTTCTCTTACC
LRR-1-NbS00005377g0007	AGTGGGAGTATACCTGACAG	ATTGATACAGAGGCCAAGTTCAG
LRR-2-NbS00007194g0113	TAGTTACAACTGCGGTCGATG	TCCAGATCCAAACATTCCACG
preoxidase-2-NbS00013071g0001	AGGCTATTCAATTTCAACTCCAC	CTTGTAGAAGCATCGGTCCAC
Psb-3-NbS00027134g0004	ATCCAGAGCAAGACATACATG	ATCAAGAGTGTAGGTTAAGCG
NBC-3-NbS00003711g0003	TCTACCAGAAATTGAGGACCG	ATTCAATTGCGTGGGGTGAGC
HSFP-NbS00043262g0009	GGAGGAGGAATGTTGGTCAAAGC	CACATTTATCCAAGTATTGCTGG
Rh-1-NbS00007177g0002	AGGAGCTCAACTAAGTGATGTG	ACCTAAGTCATTATCTGTGTTC
Ser-p-3-NbS00032762g0006	AGGCGATGGTACTATTGTTTC	ACATCAGCAGAGAAATAATGTGC
Psbp- 3-NbS00027024g0003	TGAAGCCATTGTCCTCCATC	TAGGAGAAGTAGGAAGGTAAG

F, Forward Primer; R, Reverse Primer

## Results

### Symptoms and molecular characteristics of NS3-transgenic *N. benthamiana* after infection with RSV

**Fig. 1:**
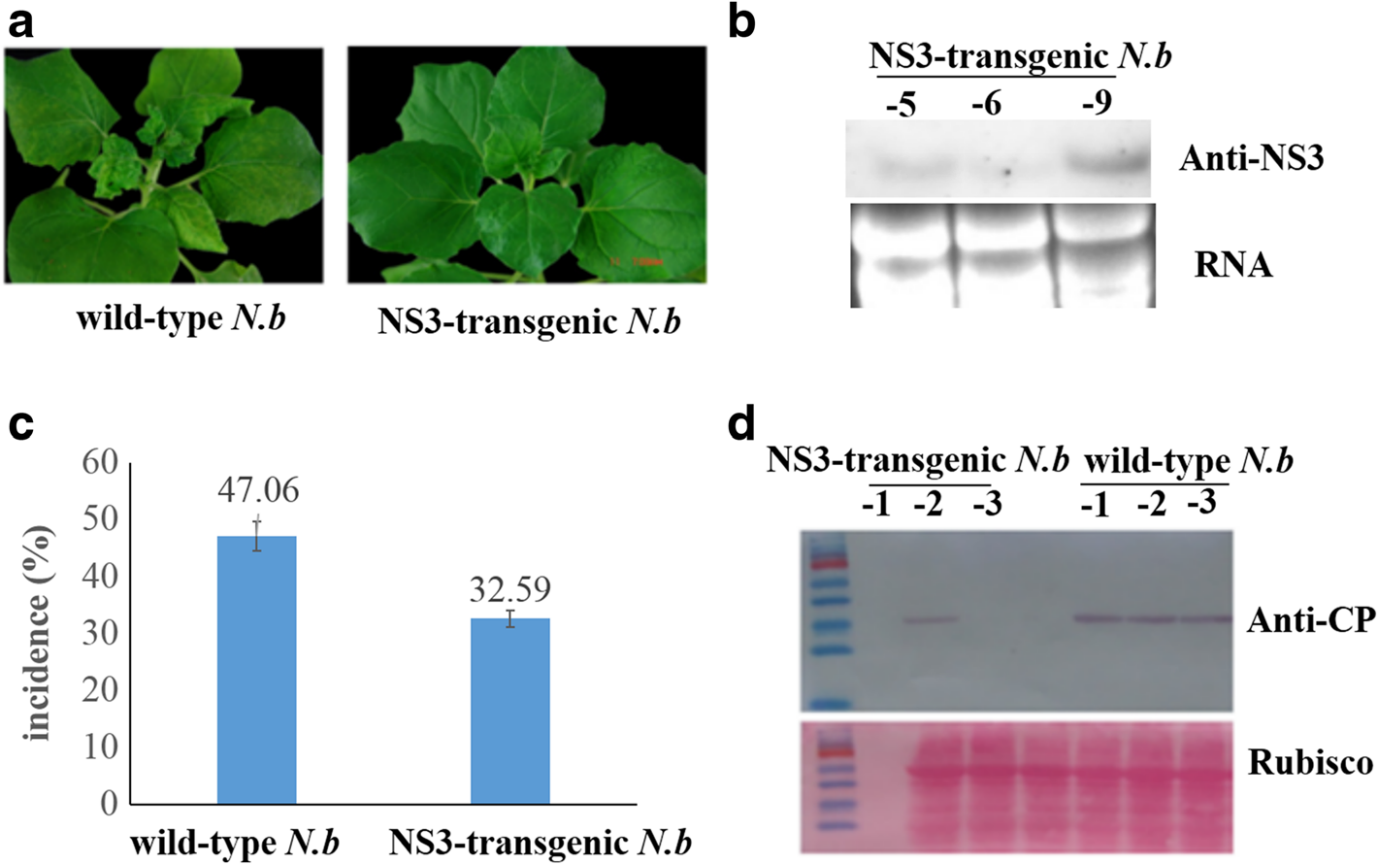
*Rice stripe virus* (RSV) infection in *Nicotiana benthamiana* (*N.b*). **a**. The symptoms of wild-type *N.b* and NS3-transgenic *N.b* inoculated with RSV; **b**. The stability of NS3 expression in transgenic *N.b* as assessed by northern blot analysis. **c**. Incidence of RSV in wild-type *N.b* and NS3-transgenic *N.b* as assessed by ELISA. **d**. RSV accumulation in NS3-transgenic *N.b* and wild-type *N.b* were estimated by western blot analysis with RSV CP-specific antibody (the Rubisco protein level served as a loading control)

We investigated the stability of NS3 expression in transgenic *N. benthamiana* by northern blot analysis, and the results showed that NS3 was transcribed in plants from different transgenic lines (NS3–5, NS3–6, and NS3–9) (Fig. 1b). To examine the susceptibility of transgenic plants to RSV, NS3-transgenic *N. benthamiana* plants and wild-type *N. benthamiana* plants were inoculated with RSV. Twenty-one days post-inoculation (dpi), viral symptoms of chlorotic and stunted stripes appeared on the leaves of wild-type *N. benthamiana*, but not NS3-transgenic *N. benthamiana* (Fig. 1a). RSV infection rates among plants were determined by enzyme-linked immunosorbent assay (ELISA). The results showed that only 35.29% of NS3-transgenic *N. benthamiana* plants were infected with RSV, compared to 47.06% of wild-type *N. benthamiana* plants (Fig. 1c). Western blot analysis of RSV accumulation in inoculated symptomatic *N. benthamiana* plants showed that the RSV titer in wild-type *N. benthamiana* plants was higher than in NS3-transgenic *N. benthamiana* plants (Fig. 1d), suggesting that NS3 is associated with plant disease symptom development.

### Overview of transcriptome sequencing and analysis of DEGs

To explore the transcriptional responses of the transgenic *N. benthamiana* to RSV, RNA from two control (CK) *N. benthamiana* plants and three NS3-transgenic *N. benthamiana* plants was used to construct five cDNA libraries. After adaptor sequence trimming and removing low quality reads, clean reads were obtained from four libraries of “NS3-transgenic” and “CK” samples. In total, 43.64 Gb of clean data with Q30 content > 85% were available for subsequent analysis (Table 2). Clean reads were mapped to the *Solanaceae* reference genome (https://solgenomics.net) using bowtie software allowing for a 2-bp mismatch. The results are shown in Table 3. The proportion of mapped gene numbers to reference gene numbers exceeded 75% in these five libraries (Table 3), indicating that our RNA-seq data were sufficient for subsequent gene expression analysis.

**Table 2:** Summary of clean reads

Sample	BMK-ID	Clean reads	Clean bases	GC Content	% ≥ Q30
CK⑤	T01	32,643,302	9,670,982,040	44.75%	85.53%
CK④	T02	31,189,890	9,225,908,582	44.60%	86.44%
NS3–5①	T03	28,561,385	8,435,736,918	44.63%	85.05%
NS3–6④	T04	25,934,242	7,723,069,786	43.81%	92.15%
NS3–9④	T05	28,825,368	8,589,301,830	43.65%	92.14%

Samples: the name and the number of samples; BMK-ID: the number of samples in BMK, Clean bases: the sum of bases; GC content: G and C bases account for percentage of all bases;≥Q30%, the percentage of greater than or equal to 30 bases

**Table 3:** Summary of sequencing data

BMK-ID	Total reads	Mapped reads	Uniq Mapped reads	Multiple Map reads
T01	65,286,604	50,211,245(76.01%)	45,993,222(70.45%)	4,218,023(6.46%)
T02	62,379,780	48,560,734(77.85%)	44,175,401(70.82%)	4,385,343(7.03%)
T03	57,122,770	42,907,734(75.11%)	39,761,682(69.61%)	3,146,052(5.51%)
T04	51,868,484	41,051,463(79.15%)	38,702,289(74.62%)	2,349,174(4.53%)
T05	57,650,736	45,691,280(79.26%)	43,190,448(74.92%)	2,500,832(4.34)

Total clean reads: the raw data after sequencing

Unique mapped reads: the high quality clean reads that can be mapped to the *Solanaceae* genome

**Fig. 2:**
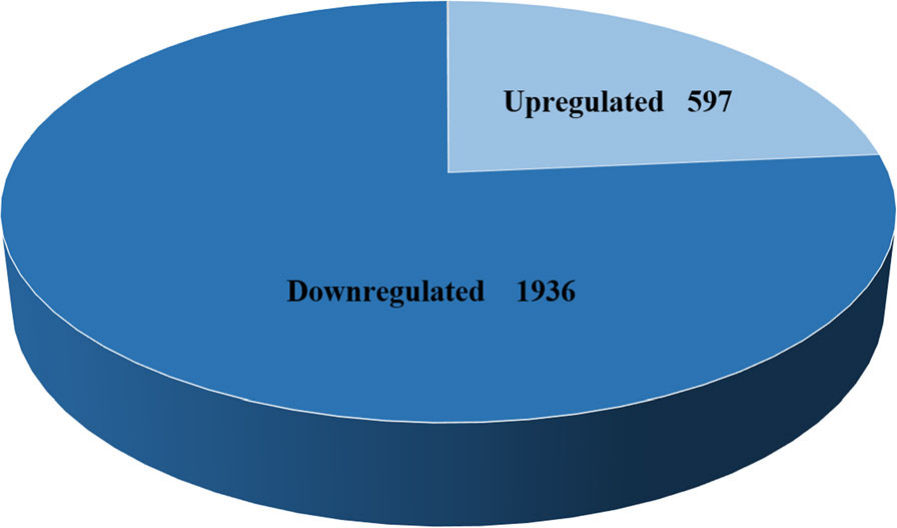
Diagram depicting the distribution of DEGs. In total, 2533 differentially expressed genes DEGs (*P* < 0.05) were identified in NS3-transgenic *N. benthamiana* and *N. benthamiana* leaf tissue, including 597 upregulated genes and 1936 downregulated genes

To characterize *N. benthamiana* candidate genes that responded to NS3, we analyzed five transcriptome profiles. First, the expression level of each gene was normalized as clean reads per kilobase of exon model per million mapped reads (RPKM). Then, DEGs were identified by comparing genes expressed in transgenic *N. benthamiana* plant samples with those from CK plants with the stringent criteria of a false discovery rate (FDR) < 0.001 and |log2Foldchange***|*** > 1. In total, 2533 DEGs were identified in these five transcriptome profiles, including 597 upregulated genes and 1936 downregulated genes (Fig. 2).

These results showed that the majority of DEGs were downregulated in NS3-transgenic *N. benthamiana* compared with control plants, suggesting that NS3 affected the expression of endogenous genes.

### GO and KEGG pathway enrichment analysis

**Fig. 3:**
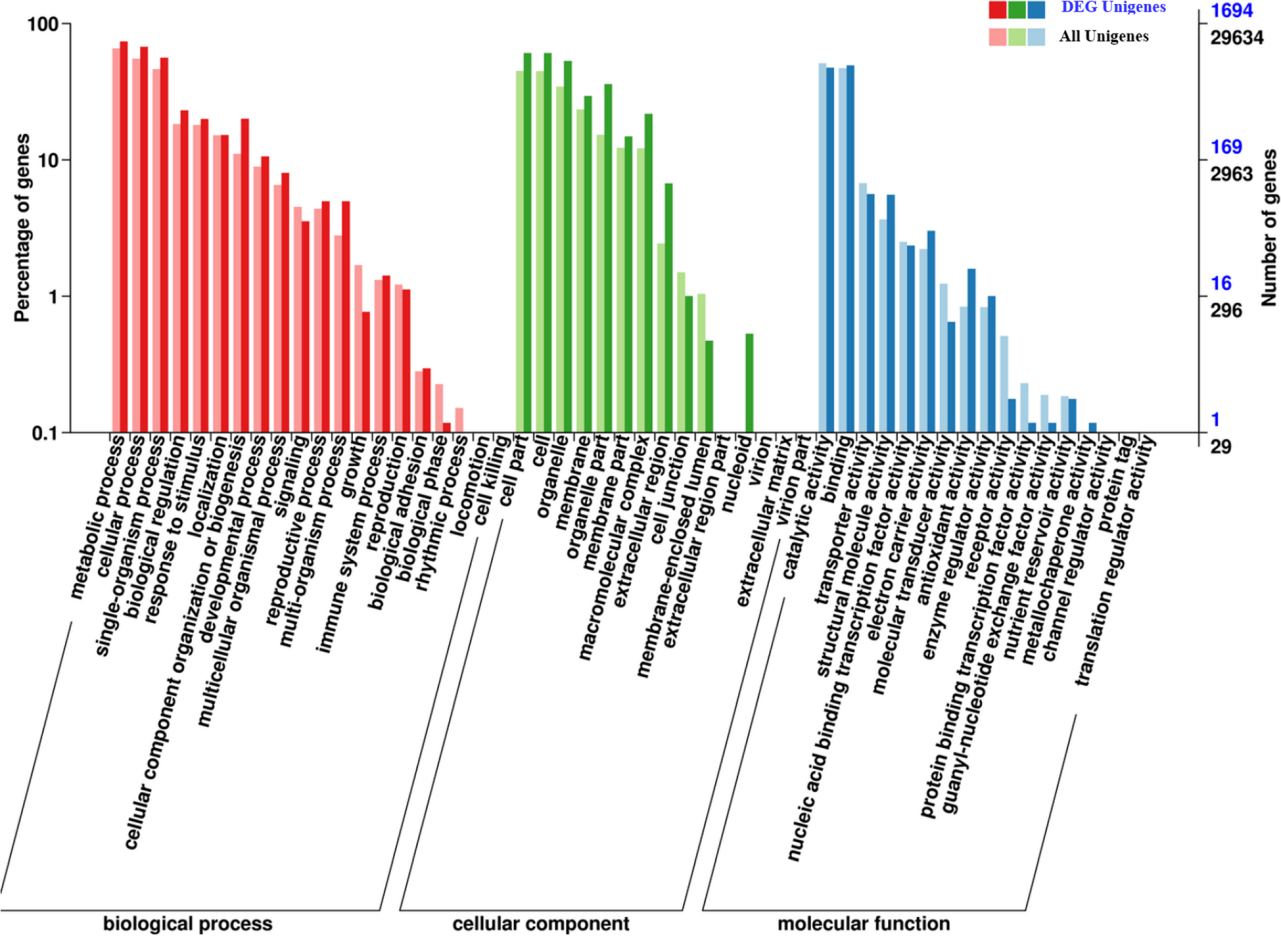
Functional distribution of DEGs in NS3-transgenic *N. benthamiana* plants at the 6th leaf stage. Left vertical coordinate represents the percentage of genes; right vertical coordinate represents the number of genes

In total, 2533 DEGs were assigned to various functional categories following the GO functional classification scheme (Fig. 3). Based on their putative functions, the DEGs were classified into three GO categories (biological process, cellular component, and molecular function), containing 43 GO terms. The three most highly represented GO terms under the biological process category were “metabolic process,” “cellular process”, and “single-organism process”. The five most abundant terms in the cellular component category were “cell part,” “cell,” “organelle,” “membrane”, and “organelle part”. The most abundant GO terms in the molecular function category were “catalytic activity” and “binding”.

**Fig. 4:**
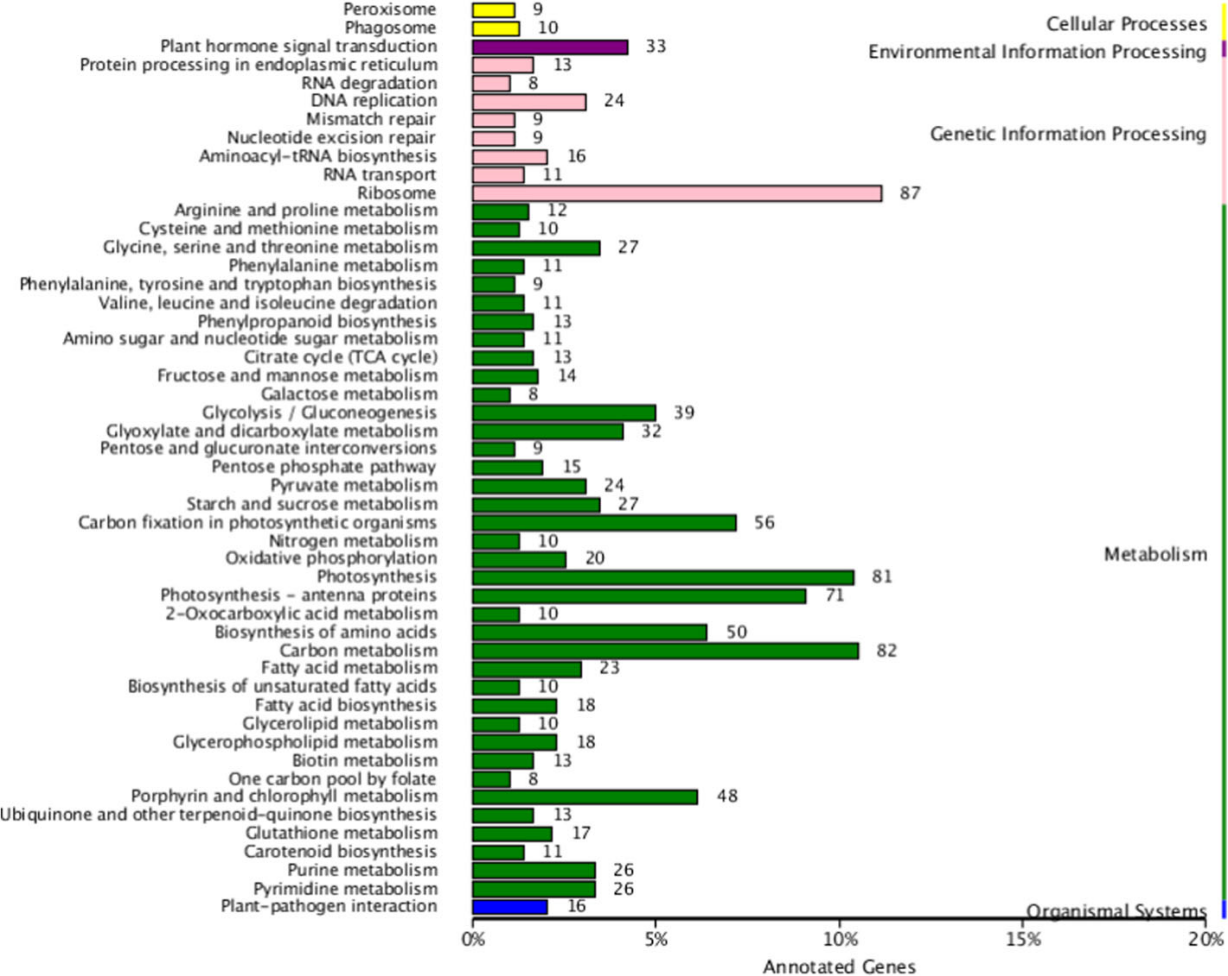
KEGG categories of DEGs in NS3-transgenic *N. benthamiana* plants at the 6th leaf stage. Left vertical coordinate represents the type of KEGG metabolism pathway; right vertical coordinate represents biological process; horizontal axis shows the percentage of annotated genes

**Fig. 5:**
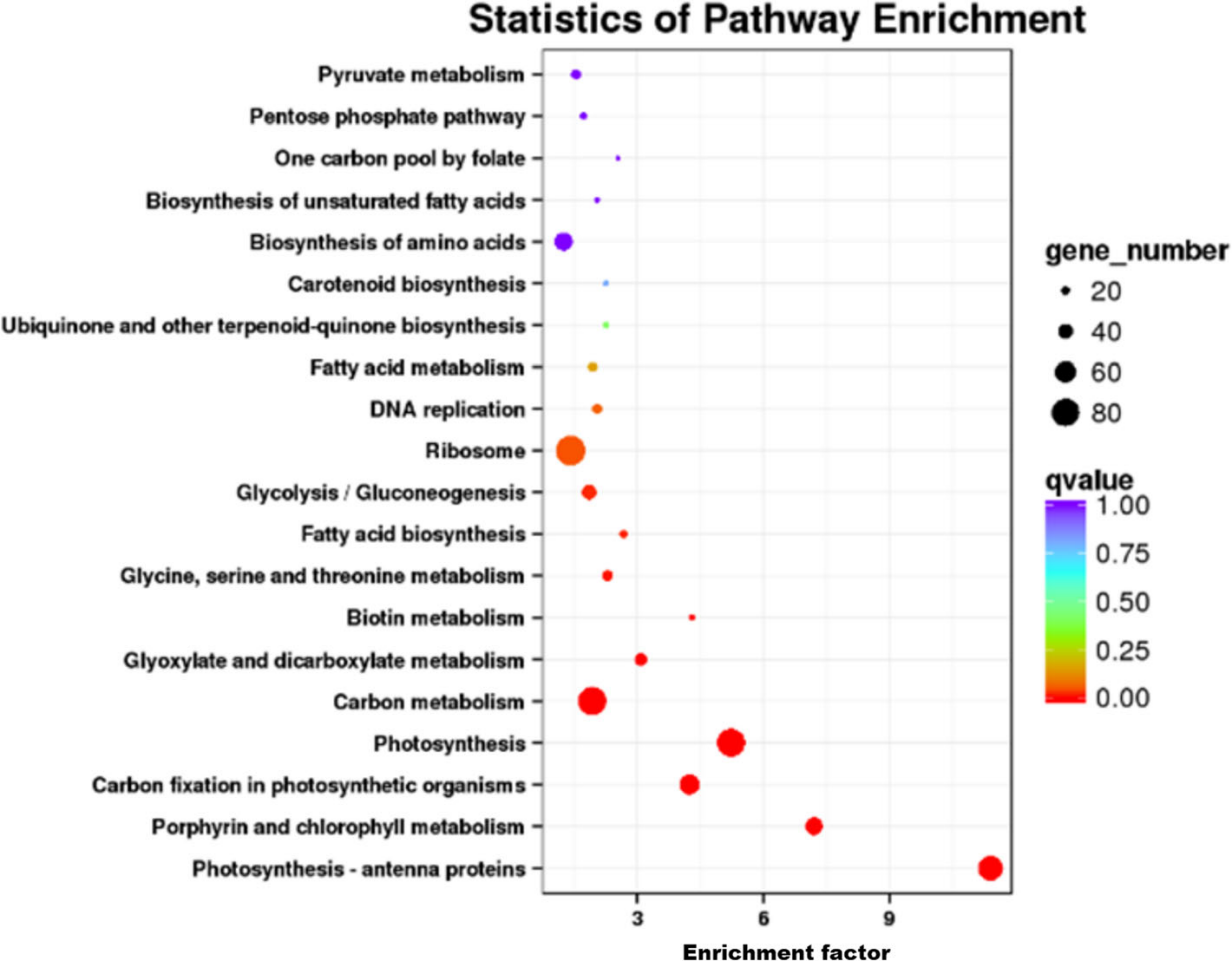
Results of pathway enrichment for DEGs in NS3-transgenic *N. benthamiana* plants at the 6th leaf stage. Spot areas indicate numbers of genes; different colors indicate q values

We conducted KEGG classification of the DEGs. The 2533 DEGs were classified into five groups (cellular processes, environmental information processing, genetic information processing, metabolism, and organismal systems) related to 50 KEGG pathways (Figs. 4 and 5). The four most common pathways were “ribosome,” “carbon metabolism,” “photosynthesis”, and “photosynthesis-antenna proteins”. There were over 70 DEGs in each of these pathways.

### Validation of transcriptome data by RT-qPCR

**Fig. 6:**
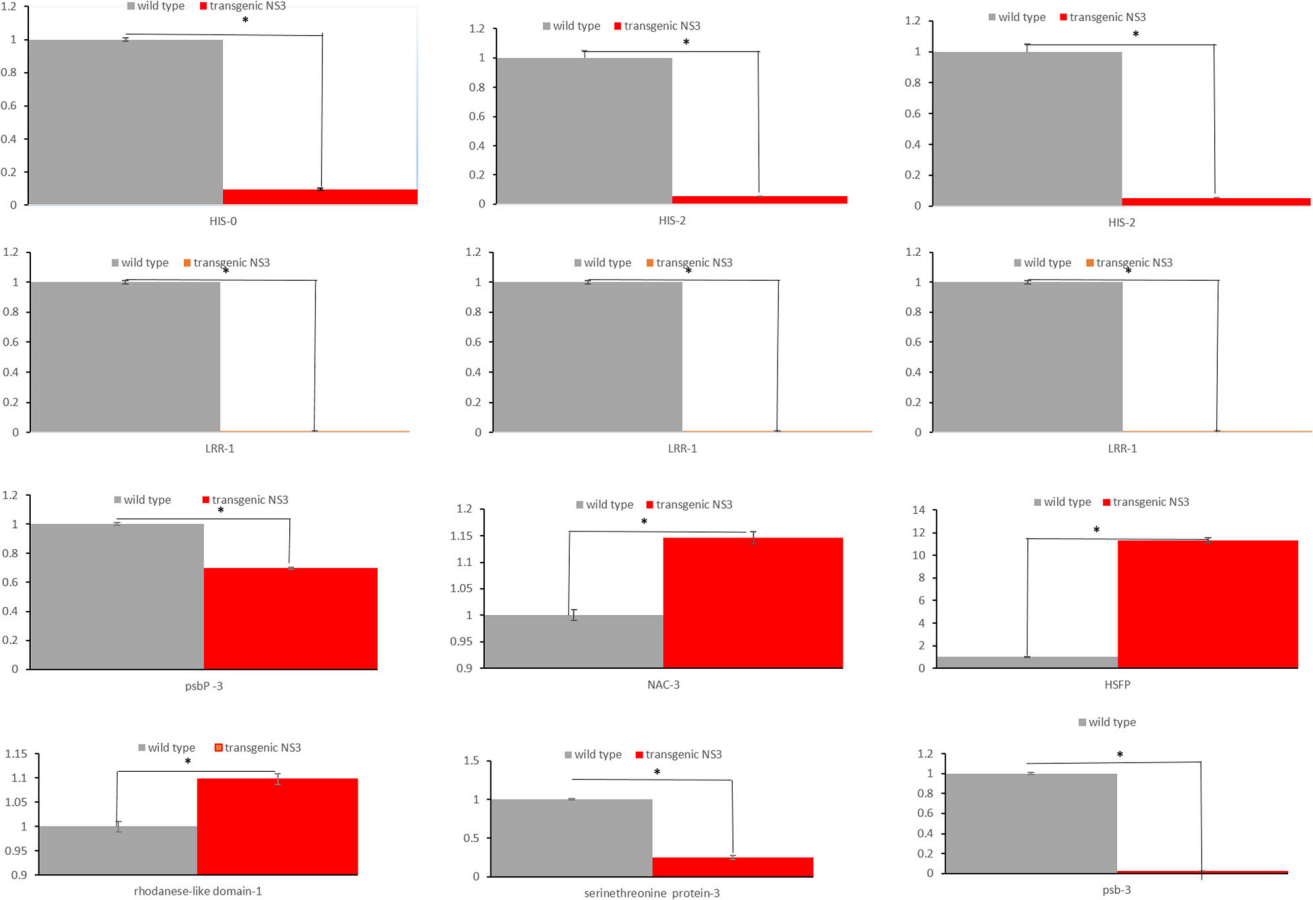
Quantitative real-time PCR (RT-qPCR) validation of DEGs. The ubiquitin gene was used as an internal control. Error bars represent the standard deviation of the RT-qPCR signals (*n* = 3). Asterisks indicate statistically significant differences compared with the control (*P* < 0.05)

We used RT-qPCR to verify the RNA-seq data (primers listed in Table 1). Genes were chosen from among DEGs in the most common pathways (ribosome and photosynthesis). The results indicated that all gene expression patterns were consistent between RT-qPCR analysis and RNA-seq analysis (Fig. 6).

## Discussion

VSR is a determinant of viral pathogenicity. Recombinant virus co-expressing heterogeneous VSR is associated with increased virulence and more severe symptoms in host plants. However, our previous research showed that NS3-transgenic rice exhibited enhanced resistance to *Magnaporthe oryzae* [18]. The expression of some VSRs as transgenes in plants also induced developmental defects in vegetative and reproductive organs [24–26]. Conversely, in this study we found that NS3-transgenic *N. benthamiana* plants not only had the normal phenotype, as observed with transgenic *N. benthamiana* expressing P19 from TBSV and P25 from *Potato virus X* (PVX) [25, 27], but also exhibited increased resistance to RSV, as observed with transgenic *N. benthamiana* expressing HC-Pro from TEV [12]. The underlying mechanism for this resistance requires further investigation, but is presumably related to NS3 activity, possibly in the nucleus [8]. Primary-miRNAs (pri-mRNAs) were differentially expressed in rice infected by RSV, and NS3 was associated with decreased accumulation of pri-miRNAs; however, the mechanism by which NS3 influences pri-miRNA expression levels remains unclear [23].

Several reports indicated that VSRs have the ability to induce upregulated expression of genes related to the plant responses to biotic or abiotic stress, in addition to their pathogenic roles during viral infection [27, 28]. For example, expression of CMV VSR 2b protein in transgenic plants conferred increased drought tolerance [28]. Expression of the PVX-specific P25 silencing suppressor in transgenic tobacco plants caused upregulation of 138 transcripts belonging to genes that are induced by various biotic and nonspecific stresses [27]. In this study, we found that NS3 affected the expression of endogenous genes in *N. benthamiana*. These DEGs were associated with 43 GO terms belonging to three categories, as well as four KEGG pathways. The most highly enriched GO terms were “metabolic process,” “single-organism process,” “cell part,” “membrane”, and “organelle part”. The key KEGG pathways were related to “ribosome,” “photosynthesis,” “photosynthesis-antenna proteins”, and “carbon metabolism”. Further functional analysis is required to clarify the roles of genes associated with these pathways. We suggest that NS3 has dual functions, including facilitating viral infection as a VSR and inhibiting pathogenic development as an inducer of host defense.

The fundamental unit of chromatin is the nucleosome, which is composed of approximately 146 base pairs of DNA wrapped around a histone octamer containing two copies each of histones H2A, H2B, H3, and H4. Chromatin structure influences the accessibility of transcription factors and cofactors for DNA-tempered processes. The structure and function of chromatin are regulated by multiple epigenetic mechanisms, including DNA methylation, histone modifications, adenosine triphosphate (ATP)-dependent chromatin remodeling, placement of histone variants, and regulation by non-coding RNA [29]. Since the histone tail domains are highly accessible to the nuclear environment, they constitute attractive targets for signal-activated enzymes, and may function as important links between signal transduction and gene expression [30]. A growing body of research suggests that histone modifications play vital roles in several chromatin-based processes [31]. Recent research has demonstrated that a single methylated H3K36 per nucleosome is sufficient to silence cryptic transcription in vivo. The modification of nucleosomes, including histones, may lead to changes in biological processes and the plant response to abiotic stress [32, 33]. The transgenic expression of RSV NS3 downregulated histone-related genes, indicating that NS3 could affect the nuclear environment of host cells.

Leucine-rich repeats receptor-like kinase (LRR-Like) genes represent a large and complex gene family in plants, and are involved mainly in development and stress responses. These receptors are composed of an LRR-containing extracellular domain (ECD), a transmembrane (TM) domain, and an intracellular kinase domain (KD) [34]. Transcriptome analysis predicted downregulated LRR genes in the NS3-transgenic *N. benthamiana*, although the precise functional roles of these genes remain unclear. However, it is plausible that NS3 expression affected the TM domain, which is required for RSV infection.

A newly discovered gene for a NAC domain protein in rice confers resistance to *Rice dwarf virus* (RDV) [35]. Similarly, genes related to the NAC domain were downregulated and associated with enhanced resistance in NS3-transgenic *N. benthamiana*. The functions of these genes will be verified in subsequent vector-based experiments using vectors. Upregulated genes related to heat shock protein (HSP) play a role in *Agrobacterium*-mediated plant transformation; therefore, decreased or increased HSP90 expression may affect transformation susceptibility [36]. The function of HSP may be further investigated using transformation studies.

## Conclusion

In this study, we showed that NS3-transgenic *N. benthamiana* plants not only exhibited a normal phenotype, but also possessed enhanced resistance to RSV. We investigated the influence of NS3 on gene expression using transcriptomic analysis. NS3 expression was associated with 2533 DEGs in *N. benthamiana*, most of which were downregulated. GO annotation and KEGG pathway enrichment analysis revealed biological pathways affected by NS3. These results enhance our understanding of the function of VSR in host plants.

## Abbreviations

AGO: Argonaute
CMV: Cucumber mosaic virus
DEGs: Differentially expressed genes
GO: Gene Ontology
HSP: Heat shock protein
KD: Kinase domain
LRR-Like: Leucine-Rich Repeats Receptor-Like Kinase
PTGS: post-transcriptional gene silencing
RdRp: RNA-dependent RNA polymerase
RDV: Rice dwarf virus
RISC: RNA-induced silencing complex
RSV: Rice stripe virus
RT-qPCR: Real-time quantitative PCR
TBSV: Tomato bushy stunt virus
TEV: Tobacco etch virus
TM: Transmembrane domain
TYMV: Turnip yellow mosaic virus
VSR: Viral suppressor of RNA silencing
